# Supplementary material for: Lab2Field transfer of a robotic raspberry harvester enabled by a soft sensorized physical twin
Source: Commun Eng. 2023 Jun 23;2:40. doi: 10.1038/s44172-023-00089-w (PMC10955996; doi:10.1038/s44172-023-00089-w)
Supplement: Supplementary file 2 — Supplementary Material [file 44172_2023_89_MOESM2_ESM.pdf]

Supplementary Material

Supplementary Note 1: Closing the reality gap of the physical twin

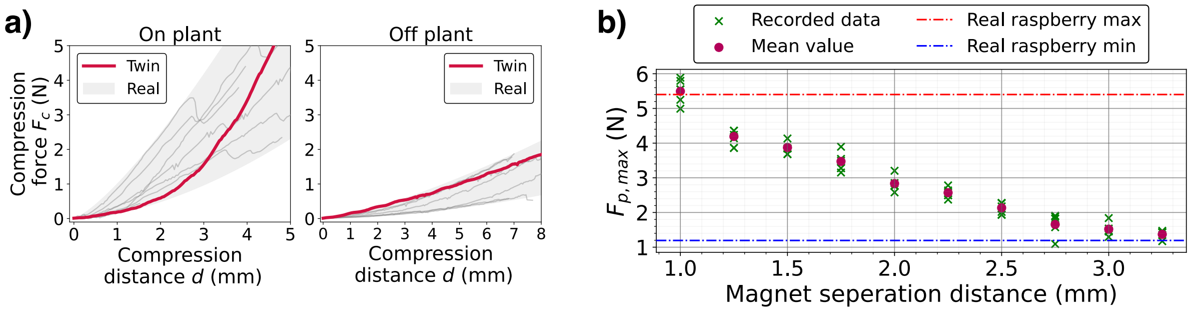

Figure S1. Plots showing k_{on}, k_{off}, and $F_{p,\max}$ of the physical twin match the real fruit.
a: Compression force vs displacement plot for the physical twin compared to real raspberries on and off the plant; highlighting k_{on} and k_{off} are matched with the plant. b: Maximum pulling force $F_{p,\max}$ of the real fruit and the physical twin. By adjusting the magnet height in the twin, $F_{p,\max}$ can be varied. For each setting five trials were conducted, and the error bars indicate one standard deviation. Data in both a and b are from [34]

Fig. S1a shows the force-displacement plot of the compression force (F_c) of the physical twin, on and off the plant. This was compared to the compression of 14 exemplar real raspberries also measured on and off the plant. The compression force of the physical twin lies within the experimentally measured values of the real fruit. Note, the stiffness of the physical twin off the plant on the stiffer end of the bound of the real fruit. Fig. S1b shows maximum pulling force $F_{p,\max}$ of the physical twin is within the bounds of the real fruit (through the same 14 exemplar raspberries) and can be tuned within this bound. The maximum and minimum value of $F_{p,\max}$ measured on the real fruit is given by the blue and red dotted lines. By tuning the magnet separation, the value of $F_{p,\max}$ is tuned within this range. The standard deviation is low, showing high repeatability, and the response is linear with distance. Hence, the physical twin can be used to span a range of different raspberry conditions, reflecting varying ripeness or other factors.

Supplementary Note 2: Result of the automatic tuning of F_c^1
Fig. S2 summarizes the tuning of F_c^1. The tuning is performed for 15 iterations for each of the pulling force setting for the physical twin For all force settings, the value of F_c^1 begins at 3.43N (350gf) and gradually converges. The final value for F_c^1 is used as the setpoint for each pulling force when testing in the field.

Supplementary Note 3: Robot system diagram
Fig. S3 describes the system diagram of the raspberry harvesting robot. The main components are the gripper, mobile manipulator, and a control computer. The control computer is operated by a human, and runs all the software required to interface with the gripper and mobile manipulator, controllers described in this paper, and data collection system.

Supplementary Note 4: Harvesting result categories
The raspberry harvested by the robot is categorized into four categories by inspection. The descriptions and names of the four categories are shown in Table S1.

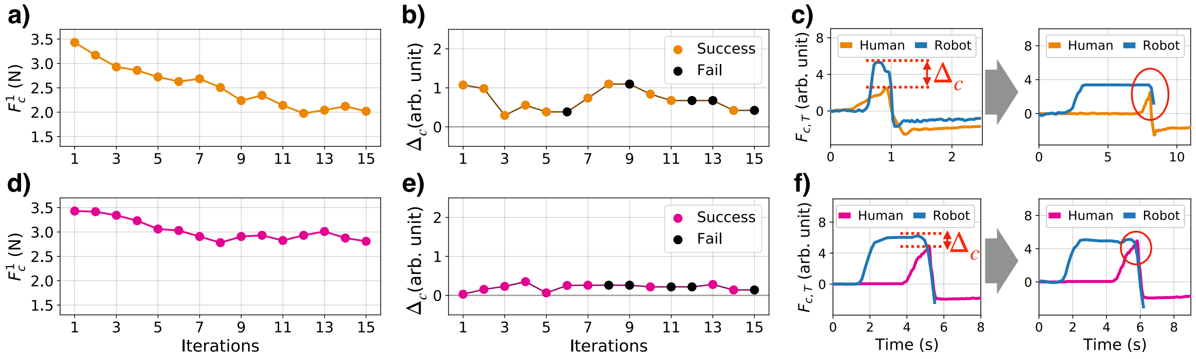

Figure S2. Automatic controller tuning for the Medium and High pulling force setting on the physical twin.
The change in F_c^1 over the 15 iterations for the Medium and High pulling force settings (a and d). How the error Δ_c to be minimized varies over the 15 iterations with indication of a failed harvest for the Medium and High pulling force settings (b and e). The compression force experienced by the physical twin before and after controller tuning for the Medium and High pulling force settings (c and f).

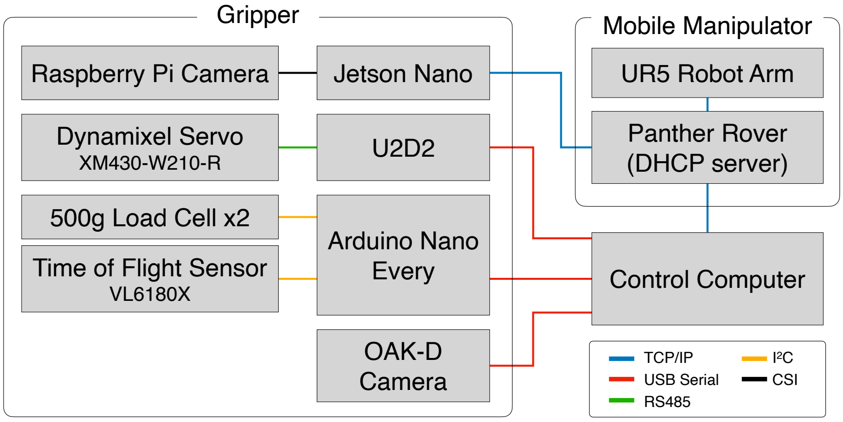

Figure S3. System diagram of the raspberry harvesting robot.

Table S1. Descriptions of the harvesting outcome categories

Category	Description
No harvest	The fruit is fully or partially on the plant after the harvesting process
Damage	The fruit is fully off the plant, but there is clear damage (e.g.: large rips, crushing)
Minor Damage	The fruit is fully off the plant. Some damage is visible but the fruit is intact (e.g.: some squashing)
No Damage	The fruit is fully off the plant. No apparent damage is visible and comparable to a human harvest
