# Supplementary material for: Lab2Field transfer of a robotic raspberry harvester enabled by a soft sensorized physical twin
Source: Commun Eng. 2023 Jun 23;2:40. doi: 10.1038/s44172-023-00089-w (PMC10955996; doi:10.1038/s44172-023-00089-w)
Supplement: Supplementary file 3 — Description of Additional Supplementary Files [file 44172_2023_89_MOESM3_ESM.pdf]

Description of Additional Supplementary Files

File Name: Supplementary Movie S1

Description: Supplementary Movie S1 shows the automatic controller tuning process. In this video, the robot harvests the physical twin in the lab. Two camera angles of the setup is given alongside live data streams of the robot and the twin's sensor readings.

File Name: Supplementary Movie S2

Description: Supplementary Movie S2 shows the full robotic pipeline deployed on the physical twin in the lab. This includes the visual servoing and the harvesting. During the visual servoing, the camera frame from the robot is shown alongside two camera views of the robot.

File Name: Supplementary Movie S3

Description: Supplementary Movie S3 shows the full robotic pipeline deployed on real raspberries in the field. The robot harvesting three different raspberries are shown.
